# Gene–Environment Interactions on Body Fat Distribution

**DOI:** 10.3390/ijms20153690

**Published:** 2019-07-27

**Authors:** Xiang Li, Lu Qi

**Affiliations:** 1 Department of Epidemiology, School of Public Health and Tropical Medicine, Tulane University, New Orleans, LA 70112, USA; 2 Department of Nutrition, Harvard T.H. Chan School of Public Health, Boston, MA 02115, USA

**Keywords:** gene–environment interaction, body fat distribution, obesity

## Abstract

The prevalence of obesity has been increasing markedly in the U.S. and worldwide in the past decades; and notably, the obese populations are signified by not only the overall elevated adiposity but also particularly harmful accumulation of body fat in the central region of the body, namely, abdominal obesity. The profound shift from “traditional” to “obesogenic” environments, principally featured by the abundance of palatable, energy-dense diet, reduced physical activity, and prolonged sedentary time, promotes the obesity epidemics and detrimental body fat distribution. Recent advances in genomics studies shed light on the genetic basis of obesity and body fat distribution. In addition, growing evidence from investigations in large cohorts and clinical trials has lent support to interactions between genetic variations and environmental factors, e.g., diet and lifestyle factors, in relation to obesity and body fat distribution. This review summarizes the recent discoveries from observational studies and randomized clinical trials on the gene–environment interactions on obesity and body fat distribution.

## 1. Introduction

The past few decades have seen a rapid increase in the prevalence of obesity in the United States and worldwide [1,2]. Results from the recent Global Burden of Disease (GBD) study, which assembled data from 195 countries, indicate that the prevalence of obesity, defined as body mass index (BMI) greater than 30 kg/m^2^, has more than doubled since 1980 [3]. Estimates from the 2015-2016 National Health and Nutrition Examination Survey (NHANES) indicate that 31.8% of U.S. adults aged 20 and over are overweight, and 39.8% are obese, with 7.6% with severe obesity. Notably, abdominal obesity, measured by waist circumference, has been increasing steadily from 99.1 (SD: 0.6) cm for men and 92.2 (0.9) cm for women in 1999–2000 to 102.1 (0.8) cm for men and 98.0 (0.7) cm for women in 2015–2016 among U.S. adults [4]. Obesity is a common risk factor for various chronic diseases, including type 2 diabetes, cardiovascular disease, certain cancers, and fertility complications [3,5,6].

BMI is a widely used proxy to estimate the overall adiposity in epidemiological studies as well as clinical practice. However, BMI cannot differentiate fat and lean mass, nor the distribution of body fat. In epidemiological studies, body fat distribution is associated with risks of type 2 diabetes, coronary heart disease, and all-cause mortality, independent of overall adiposity as measured by BMI [7,8,9,10,11]. Among individuals equally overweight or obese, those with higher central adiposity showed a greater risk of developing cardiometabolic disorders [9,12,13,14,15,16]. In contrast, those with higher gluteal adiposity exhibited lower risks of type 2 diabetes, hypertension, dyslipidemia, and mortality [17,18,19]. Commonly used anthropometric measures for body fat distribution include waist circumference (WC), waist-to-hip ratio (WHR), and WHR adjusted for BMI (WHRadjBMI). Such anthropometric parameters are informative and easy to collect. Other more refined phenotypic measurements, such as magnetic resonance imaging (MRI)- and computed tomography (CT)-based measurements, can provide incomparable insight of the amount of adipose tissue residing in particular depots, although the application of these measurements in large-scale studies is challenging.

Since 2002, rapid advances in genetic research and the application of genome-wide association studies (GWAS) have been successful in identifying genetic factors associated with various traits and diseases [20]. Although many genes have been identified to be associated with obesity and body fat distribution, there is a growing consensus that the genetic variants with modest effects discovered by GWAS do not account for a large proportion of estimated heritability of obesity and body fat distribution. Such missing heritability may at least partly be explained by the gene–environment interaction, which is not explicitly modeled in GWAS [21,22]. The increasing body of studies on gene–environment interactions in relation to obesity lends great support to the hypothesis above. In addition, the recently emerged omics studies, such as metabolomics and microbiome, are generating novel data regarding other non-genetic factors affecting the development of obesity and body fat distribution. The purpose of the current review is to summarize the recent findings of gene–environment interactions in relation to obesity and body fat distribution from observational studies and randomized clinical trials. The review also briefly discusses other omics studies on body fat distribution, challenges in this field, and future directions.

## 2. Genetics of Obesity and Body Fat Distribution

Many studies have indicated a genetic component in determining obesity and body fat distribution [23,24,25]. Familial aggregation analysis, including twin and adoption studies, consistently estimate the heritability of BMI to be approximately 40–70% [26,27,28,29,30]. In addition, there has been compelling evidence supports that the genetic control of regional fat deposition distinct from the overall adiposity. For example, the heritability of WC and WHR is estimated to be 30–45%, even after adjusting for BMI [16,30,31,32]. Visceral adipose tissue (VAT) and subcutaneous adipose tissue (SAT) measured by CT scans are also demonstrated to be 36% and 57% heritable, respectively [33].

**Figure 1:**
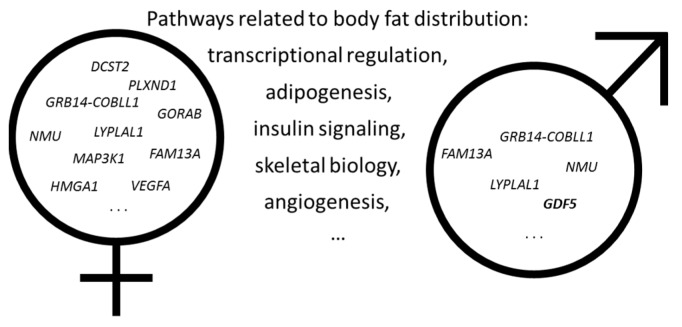
Sexual dimorphism in genetically determined body fat distribution and related pathways. This figure shows selected GWAS-identified genes and pathways displaying sexual dimorphism in relation to body fat distribution. The genes and pathways included are incomplete, only for illustration purpose.

**Figure 2:**
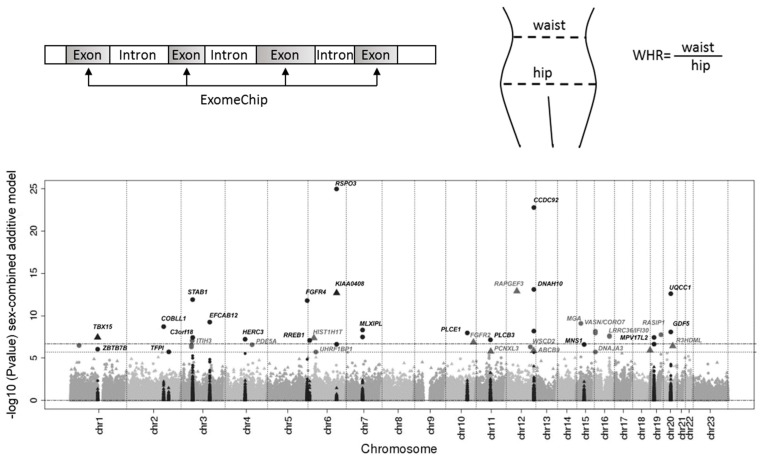
GWAS of body fat distribution assessed by waist-to-hip ratio adjusted for body mass index. This figure shows the coding variants identified from the most recent GWAS of body fat distribution. Manhattan plot of the all ancestry, sexes-combined, single variant, additive model analysis. The figure was modified with permission from [39]; published by Springer Nature, 2019.

With the advances in genotyping technologies, along with the advent of large-scale data, such as the UK Biobank, China Kadoori Biobank, and All of US, researchers have successfully identified hundreds of specific genetic variants associated with complex traits, including obesity and body fat distribution. The vast majority of these genetic variants were identified for BMI and WHR among European ancestry. The most recent meta-analysis of GWAS identified 941 independent single nucleotide polymorphisms (SNPs) associated with BMI [34]. Collectively, those genome-wide significant SNPs explain ~6% of the variance of BMI [34]. Interestingly, many of these identified loci were found to be heavily involved in pathways of the central nervous system, such as regulation of appetite and food intakes [35]. Unlike overall adiposity, the genes related to regional fat distribution shows a high degree of sexual dimorphism (Figure 1). For example, Shungin et al. identified 49 loci associated with WHRadjBMI, of which 20 showed high sexual dimorphism [16]. Nineteen of the 20 loci displayed a stronger effect in females, while only one genetic variant located on *GDF5* gene was stronger in males (Figure 1) [16]. In the recent GWAS meta-analysis of body fat distribution, measured by WHRadjBMI, 346 loci were identified, with approximately one-third of them stronger among women than men [36]. In another GWAS of body fat distribution, measured by segmental bio-electrical impedance analysis (sBIA), 98 independent SNPs were identified, and 37 SNPs exhibited stronger association in females than in males [37]. Most of the previous GWAS identified common, non-coding variants, from which pinpointing the causal genetic variants remains challenging. A recent meta-analysis of exome-targeted genotyping data identified 14 rare and low-frequency coding variants associated with BMI [38]. The results show a 10-fold larger effect of rare variants than of common variants; the largest effect was found in carriers of *MC4R* stop-codon (p.Tyr35Ter, minor allele frequency = 0.01%), weighing about 7 kg heavier than non-carriers [38]. Justice et al. analyzed coding variants from ExomeChip data in up to 476,546 individuals and identified 15 common and nine low frequency of rare coding novel variants significantly associated with WHRadjBMI (Figure 2) [39]. Similar to other prior genetic studies of body fat distribution, the authors observed sexual dimorphism in the genetic architecture of WHRadjBMI, with 16 out of 19 coding variants displaying larger effect in women than in men [39].

The gold standard methods for assessment of body fat distribution include CT and MRI. However, the sample sizes of studies using these gold standard measurements of body fat distribution were largely limited due to the high cost [40,41,42,43,44]. For example, the currently largest GWAS (*N* = 10,577) on CT measured SAT and VAT identified a genetic variant (rs1659258) near *THNSL2* gene associated with VAT only among women [40]. In addition, the body fat distribution-associated genetic variants were found to be enriched in pathways involved in adipocyte biology [45]. Interestingly, a recent GWAS identified six novel loci associated with a composite body shape phenotype defined by a combination of BMI, height, weight, waist and hip circumferences, and WHR [46]. Such findings suggest that, for complex traits such as obesity and fat distribution, leveraging multiple traits may lead to novel insight into the biological pathways.

## 3. Gene–Environment Interaction on Obesity and Body Fat Distribution in Observational Studies

The continual increase in obesity and obesity-related disorders has been paralleled with dramatic changes from a “traditional” to “obesogenic” living environment, featured by the abundance of palatable, energy-dense diet, reduced physical activity, prolonged sedentary time, deprived sleep, and the shift in cultural background [47,48]. Obesogenic diet and lifestyle are believed to be the major driving forces for the obesity epidemic. However, it has long been noted that substantial inter-individual variability exists in response to diet/lifestyle modifications, and inherent factors such as genetic makeup may at least partly account for such variability [21,48,49,50,51]. Indeed, there has been a growing body of studies on gene–environment interactions in relation to obesity and body fat distribution, lending support to the hypothesis that missing heritability is at least partly explained by gene–environment interactions [48,52,53,54]. To investigate the ways in which genetic variants interact with environmental factors may provide new insight into the biology of obesity and body fat distribution, as well as develop personalized intervention strategies to reduce the risk of obesity-related disorders.

Numerous observational studies specifically investigated the interactions of obesity or body fat distribution associated SNPs or genetic risk scores (GRS) with nutrients [55,56,57,58,59,60,61], foods [55,62,63], dietary patterns [63,64,65,66,67], physical activity and other lifestyle factors [61,67,68,69,70,71]. In a previous cohort study, which included 334 female twins (57.7 ± 6.7 years), it was found that, when carrying a low genetic risk of abdominal fat, women in the highest tertile of polyunsaturated fat intakes had about 50% less central abdominal fat than those in the lowest tertile of intakes [61]. No association between polyunsaturated fat and central abdominal fat was observed among those with high genetic risk [61]. The interactions between genetic factors and environmental factors have been observed at a very young age. In a retrospective study among 2,346 children, a significant interaction was found between mono- and polyunsaturated fatty acids intake at the age of seven and the obesity GRS (constructed by eight genetic variants with known associations with BMI in children) on body fat mass index (FMI) at the age of nine; the GRS was positively associated with FMI among children with inadequate intake of mono- and polyunsaturated fatty acids, while among those with appropriate intake of mono- and polyunsaturated fatty acids, no association was observed [60]. In three large prospective cohorts (the Nurses’ Health Study, Health Professionals Follow-up Study, and Women’s Genome Health Study), we observed consistent interactions between obesity GRS and sugar-sweetened beverages (SSB) on BMI across three cohorts, whereby the genetic associations increased stepwise with increasing SSB intakes [72]. Such interactions were also replicated by two Swedish cohorts [73,74]. Olsen et al. identified significant interactions between four different obesity-related GRSs and soft drink consumption on adiposity traits [74]. The study included a total of 4,765 individuals from the Danish part of MONICA (Monitoring Trends and Determinants of Cardiovascular Disease) Study, part of the DCH (Diet, Cancer, and Health) Study, and the Inter99 study [74]. The results show that each additional risk allele of the BMI GRS was associated with an increase of WC of 0.05 cm/year (95% CI: 0.02, 0.09 cm/year; *p* = 0.001) per serving of soft drinks per day. A similar interaction was also observed with the complete GRS, which included 50 SNPs [74]. Nearly identical results were found when analyzing the WC adjusted for BMI as an outcome [74]. In Framingham Heart Study, with 1521 participants from the second- and third-generation cohort, a higher genetic risk score of non-alcoholic fatty liver disease was associated with increased liver fat accumulation in those who had decreased Mediterranean-style diet score (MDS) or Alternative Healthy Eating Index (AHEI), but not in those with stable or improved diet scores [75].

The genetic association with obesity-related traits could be intensified by an obesogenic environment [76]. Since foods or nutrients are not consumed in isolation, instead, it may represent a broader picture of correlated and complex food networks. In addition, diet habits and lifestyle factors are usually correlated; thus, it is important to investigate the interaction between genetic variants and overall diet pattern or lifestyle in relation to obesity and body fat distribution. In one previous study, Young et al. observed interactions between the *FTO* gene (which shows the strongest association with obesity) and various lifestyle and environmental factors, including physical activity, frequency of alcohol consumption, dietary variation, sleep duration, smoking, TV watching, and socioeconomic status on BMI among participants from the UK Biobank [67]. In a recent study, Wang et al. comprehensively examined the interactions between 77 BMI-related SNPs and three diet quality scores (AHEI-2010, the Dietary Approach to Stop Hypertension (DASH) diet score, and the Alternative Mediterranean Diet score (AMED)) on BMI and body weight in two large prospective cohorts in U.S. [66]. The study found that, during a 20-year follow-up, genetic associations with BMI was significantly attenuated with increasing adherence to healthy dietary patterns [66]. For example, four-year changes in BMI per 10 risk allele increment were 0.07 (SE: 0.02) among participants with decreased AHEI-2010 score and −0.01 (0.02) among those with improved AHEI-2010 score, which corresponds to 0.16 (0.05) kg vs. −0.02 (0.05) kg weight change [66]. Since many of the identified SNPs were located in central nervous system pathways, another study performed a similar analysis, stratifying by two GRSs related to central nervous system-related and non-central nervous system-related SNPs [63]. The authors found that the interactions on BMI were more profound for central nervous system-related GRS (*p* < 0.01 for 3 diets score) than for non-central nervous system GRSs (*p* > 0.05 for three diet scores) [63]. In another study with 18 cohorts of European ancestry (*N* = 68,317), the authors tested whether a composite dietary score representing healthy diet modifies associations of two GRSs (constructed by 32 BMI and 14 WHR-related SNPs) with obesity traits [65]. The composite dietary score captured information on self-reported intakes of whole grains, fish, fruits, vegetables, nuts/seeds (favorable) and red/processed meats, sweets, sugar-sweetened beverages and fried potatoes (unfavorable). The authors found that genetic susceptibility was slightly more pronounced in those with healthier diets [65]. Although these observations appear to counter the general hypothesis that healthy behaviors can offset risk, it is important to note that, at all levels of genetic susceptibility, the obesity traits (BMI or WHRadjBMI) were lower in participants with healthier than those with less-healthy diets [65]. Such gene–environment interactions in relation to obesity and body fat distribution were also observed between obesity-related genetic variants and physical activities, sleep, and other lifestyle factors [61,67,68,69,70,71,77,78], where the genetic association with obesity and/or body fat distribution appeared to be more pronounced among people with an unhealthy lifestyle. For example, in our previous study, we demonstrated that prolonged TV watching accentuated the predisposition to elevated BMI, whereas greater leisure-time physical activity attenuated the genetic association [68]. In a study in Norway, it was also reported that physical activity attenuated the genetic predisposition on BMI and WHR, especially among 20–40 years old adults [78]. Another study investigated whether the genetic predisposition to BMI and WC was modified by various sleep characteristics [77]. The study found that the genetic effect on adiposity (BMI and WC) appeared to be augmented by unhealthy sleep characteristics, including prolonged sleep duration, usual day napping, shift work, night shift work, and evening chronotype [77].

## 4. Genotype and Changes in Weight and Body Fat Distribution in Response to Diet/Lifestyle Interventions

Although there is mounting evidence supporting the gene–environment interactions on obesity and body fat distribution from observational studies, the inherent limitations of observational study design, such as reverse causation, biases, and confounding, restrict the ability to infer causality. In addition, in cohort studies, it is difficult to investigate the gene–environment interactions on weight loss, because participants in most of the observational cohorts gain body weight during follow-up. Therefore, it is essential to test the gene–environment interaction in randomized clinical trial settings, where the biases and confounders are largely controlled when the randomization is performed successfully with a large sample size. Interestingly, although there have been various named diet programs with or without lifestyle inventions, a recent network meta-analysis reported that there were small differences between named diets; various low-carbohydrate or low-fat diets showed similarly significant weight loss [79]. As mentioned above, inter-individual variability has long been observed in response to the diet or lifestyle interventions, and genetic makeup and its interactions with the interventions may partly account for such inter-individual differences. We conducted a series of studies on gene–diet interactions in thus far the largest and longest randomized diet intervention trial, the Preventing Obesity Using Novel Dietary Strategies (POUNDS Lost) trial [80]. Briefly, a total of 811 overweight and obese participants were randomly assigned to one of four diets with targeted percentages of energy from fat, protein, and carbohydrate [80]. At six months, participants assigned to each diet lost an average of 6 kg and began to regain weight after 12 months. By two years, weight loss remained similar across the four diets. A summary of select studies of gene–environment interactions in relation to body fat distribution among POUNDS Lost trial is shown in Table 1.

In one study, we found SNP rs1558902 in obesity gene *FTO* significantly interacted with dietary protein intake on two-year changes in fat-free mass (FFM, *p*-interaction = 0.034) and total percentage of fat mass (FM%, *p*-interaction = 0.049), as well as SAT (*p*-interaction = 0.001), VAT (*p*-interaction = 0.012), and total adipose tissue (TAT, *p*-interaction = 0.002) [81]. For example, among those who consumed a high-protein diet, changes (standard error, SE) in FFM and FM% per A allele were −0.63 (0.30) kg and −1.13 (0.41) at two years, respectively. Among those with an average-protein diet, no such significant relationships were found [81]. The results indicate that a high-protein diet might benefit those with the risk A-allele in terms of weight loss, and improvement in body composition and fat distribution. Of note, our data, together with other epidemiological and experimental studies [88,89,90], indicate that the genetic effects of *FTO* gene might be different on changes of fat mass at various depots [81]. However, evidence from a recent meta-analysis of eight randomized clinical trials conducted in overweight or obese participants shows that carrying the minor allele of *FTO* was not associated with differential changes in adiposity in response to dietary, physical activity, or drug-based weight-loss interventions [91]. The failure in the identification of the genetic modification effects in this study might be due to the diversity in study design and population characteristics in the participating trails. Large collaborations across multiple clinical trials are also needed to investigate the *FTO*–environment interactions on changes of fat distribution, with more consistent design and improved power. In another study, we observed significant interactions between a gut microbiota related *LCT* genotype and dietary protein in relation to body composition and abdominal fat distribution (all *p*-interaction < 0.05, except for changes in deep subcutaneous adipose tissue) [82]. In response to a high-protein diet, the G allele of *LCT* variant rs4988235 was associated with a change (SE) of −0.9 (0.43) in whole-body fat mass percent, −1.06 (0.58) in trunk fat percent, −0.89 (0.42) kg in SAT, −0.63 (0.27) kg in VAT, and −1.69 (0.76) kg in TAT at two years [82]. Again, our results show that interactions mainly on changes in ectopic fat distribution, but not on overall body weight. Except the interaction between dietary protein intake and genetic predisposition to obesity and body fat distribution, we also found interactions between genetic variants at several loci and dietary fat intake. For example, the T allele of *HNF1A* rs7957197 was associated with a greater reduction in weight and waist circumferences among participants who consumed a high-fat diet. Such results were replicated in another similarly designed clinical trial, the Dietary Intervention Randomized Controlled Trial (DIRECT) [87]. In another study, we observed that dietary fat intake significantly modified the relationship between a variant on the *MTNR1B* gene and changes in body weight, waist circumference, fat mass, lean body mass, total fat percent, and trunk fat percent [84]. In the low-fat diet group, increasing copy of the *MTNR1B* rs10830963 G allele was significantly associated with a decrease in weight, BMI, and WC. In the high-fat diet group, carrying the G allele was positively associated with changes in body fat [84].

## 5. Insight into the Role of the Gut Microbiota and Metabolites in Obesity and Body Fat Distribution

Gut microbiota is the microorganisms living in our intestine, which include at least 1000 different species of known bacteria with more than 3 million genes (150 times more than human genes). Growing evidence indicates that human gut microbiota plays an important role in body weight, fat mass, and metabolic diseases [82,92,93,94,95,96,97,98,99,100,101]. It has been shown that obese individuals have different intestinal microbiota composition from lean individuals, with a shift in the proportion of bacterial flora belonging to the Firmicutes relative to Bacteroidetes phyla [98,99,100]. Bacteria belonging to Firmicutes phyla are more efficient in harvesting energy from short-chain fatty acids and are related to increased weight and development of obesity [97,102,103]. Diet has a potential modifying effect on gut microbiota composition [104,105,106], suggesting the importance of investigations on interactions between gut microbiota and dietary factors. Several studies have identified a bunch of genetic variants associated with host gut microbiota [107,108,109,110]. A locus at the *NAT2* gene was found to be significantly associated with visceral adipose fat mass, indicating the potential mechanism underlying the link between the microbiome and abdominal obesity [111]. In our previous analysis, we found significant interactions between a *Bifidobacterium* abundance related genetic variant at the *LCT* gene and dietary protein intake on body composition and abdominal fat distribution [82].

Metabolomics investigates the small molecules produced by the process of metabolism [112], and thus holds promise to provide unparalleled ability in understanding the pathways of gene–environment interactions in relation to obesity and body fat distribution on the molecular level. There has been a growing number of studies investigating the relation of metabolites with body fat distribution [113,114,115,116,117,118,119]. Among various metabolites, branched-chain amino acids (BCAAs) and glutamate have been shown to be related to obesity, visceral adiposity, and other metabolic disorders [118,119,120,121,122]. Trimethylamine-N-oxide (TMAO), a gut microbiota-derived metabolite, and its precursors have also been associated with improvement in body weight, waist circumference, body fat composition, and fat distribution in response to diet interventions [123]. More recently, the role of short-chain fatty acids is starting to be appreciated [124,125,126,127,128]. A recent study found that plasma short-chain fatty acid acetate, propionate, and butyrate were positively associated with BMI, visceral and subcutaneous fat [128]. In addition, genetic studies revealed a group of genetic variants associated with circulating levels of metabolites such as BCAAs [129,130]. With such genetically determined metabolites, one can apply the gene–environment interaction analysis to investigate the effects of genetic predisposition to certain metabolite and adiposity in response to diet or lifestyle interventions. In one of our previous analyses, we examined a genetic variant determining BCAAs/aromatic amino acids ratio near *PPM1K* gene and observed significant interaction with dietary fat on weight loss and changes in insulin resistance [131].

## 6. Summary and Future Direction

Obesity and ectopic body fat distribution are determined by both genetic and environmental factors. The findings from current studies of gene–environment interactions hold great promise to give new insights into the biology underlying obesity and body fat distribution. Our current understanding about how these genetic factors interact with environmental factors, especially the underlying mechanisms, is still extremely lacking. Although we and other groups have conducted analyses on the interactions between various genetic and environmental factors in both observational and randomized clinical trial settings, the results might be subject to potential confounding, biases, reverse causation, and, particularly, and lack of replications [21,54]. Definitely, collaboration and data sharing would aid not only research on gene–environment interactions, but also other investigations. Large-scale and long-term intervention trials are needed to further explore the gene–environment interactions in relation to obesity and body fat distribution. In addition, most of the current statistical models for gene–environment interactions are too simplified. The common approach used in gene–environment interaction analysis is to test the significance of the multiplicative term in the model. Alternatively, one can also test the additive interaction by measuring the departure from the addition of each individual component’s effect size. The multiplicative model is easier to test and understand, while the additive model underpins the methods for assessing the biological interaction. However, the complex biological crosstalk between genetic factors and environmental factors in relation to obesity and body fat distribution may not be fully captured by such naive statistical models. Moreover, the measurement of environmental factors is much more complicated than that of inherited genetic factors. Thus, accurate and valid measurement tools are needed to assess the environmental factors, especially diet, physical activity, and other lifestyle factors. Although there have been various newly proposed approaches to test gene–environment interactions [132,133,134,135,136], as to which method provides the most powerful and robust result depends on several considerations. The effort towards deep phenotyping, improved analytical methods, large scale and replicable data, as well as the incorporation of functional annotations, would be necessary for the future analysis on gene–environment interactions.

In terms of application purpose, investments in understanding the gene–environment interactions in relation to obesity and body fat distribution are in pressing need to make the applications of precision nutrition practical. While the gene–environment interaction is still in the early stages, better knowledge on the human genome, microbiome, metabolomics, and other omics can facilitate the progress of precision nutrition application. To achieve the largest efficiency, individualized interventions, such as weight-loss diet, could be developed and delivered to subgroups based on their genetic background.

The epidemic of obesity and ectopic fat distribution is complicated and multifactorial, including not only solely genetic factors and environmental changes, but also the interactions between genetic makeup and environmental factors. Recent evidence from gene–environment interaction studies has demonstrated promising capabilities in revealing the missing heritability. The advance in new technologies and novel methods, along with the advent of large-scale data, provides opportunities to confirm the previous findings and further explore the mechanism underlying the development of obesity and ectopic fat distribution. Analysis combining the information from multiple omics data (genomics, proteomics, and metabolomics) and modifiable lifestyle factors on obesity and body fat distribution would be of importance in supporting precision nutrition and precision medicine.

## Figures and Tables

**Table 1:** Selected studies on gene–diet interactions on weight loss, abdominal fat distribution, and body composition in POUNDS Lost trial.

Study	Genetic Factor	Environment Factor	Major Finding
Zhang et al. [81]	Obesity-related *FTO* variant rs1558902	Dietary protein	Dietary protein significantly modified the *FTO* genotype in relation to weight loss and improvement in body composition and abdominal fat distribution
Heianza et al. [82]	Gut microbiota related *LCT* variant rs4988235	Dietary protein	In response to a high-protein diet, the G allele of *LCT* variant rs4988235 was associated with a greater reduction of whole-body fat %, trunk fat %, SAT, VAT, and TAT.
Heianza et al. [83]	Macronutrient intake related *FGF21* variant rs838147	Dietary carbohydrate/fat	Dietary carbohydrate/fat intake significant interaction with the *FGF21* genotype on 2-year changes in WC, percentage of total fat mass, and percentage of trunk fat
Goni et al. [84]	Circadian rhythm-related *MTNR1B* genetic variant rs10830963	Dietary fat	Carriers of the G allele of the *MTNR1B* genotype and low-/high-fat diet on changes in weight, BMI, waist circumference (WC) and total body fat
Mattei et al. [85]	*TCF7L2* gene variant rs12255372	Dietary fat	Significant interactions were observed for rs12255372 T allele and fat intake for changes in BMI, total fat mass, and trunk fat mass; TT carriers have more reductions in body composition when consuming a low-fat diet.
Lin et al. [86]	*NPY* variant rs16147	Dietary fat	The rs16147 T allele appeared to associate with a more adverse change in the abdominal fat deposition in the high-fat diet group than in the low-fat diet group.
Huang et al. [87]	*HNF1A* gene variant rs7957197	Dietary fat	Individuals with T allele of *HNF1A* rs7957197 have a greater decrease in body weight, WC when consuming a high-fat diet.
